# Interactome Analysis of the Nucleocapsid Protein of SARS-CoV-2 Virus

**DOI:** 10.3390/pathogens10091155

**Published:** 2021-09-08

**Authors:** Xiaoqin Zheng, Zeyu Sun, Liang Yu, Danrong Shi, Miaojin Zhu, Hangping Yao, Lanjuan Li

**Affiliations:** State Key Laboratory for Diagnosis and Treatment of Infectious Diseases, National Clinical Research Center for Infectious Diseases, Collaborative Innovation Center for Diagnosis and Treatment of Infectious Diseases, The First Affiliated Hospital, Zhejiang University School of Medicine, Hangzhou 310003, China; 11818225@zju.edu.cn (X.Z.); zeyusun@zju.edu.cn (Z.S.); Yu-liang@zju.edu.cn (L.Y.); shidr@zju.edu.cn (D.S.); zhumiaojin@zju.edu.cn (M.Z.)

**Keywords:** viral–host interactome, ribonucleocapsid, SARS-CoV-2

## Abstract

SARS-CoV-2 infection has caused a global pandemic that has severely damaged both public health and the economy. The nucleocapsid protein of SARS-CoV-2 is multifunctional and plays an important role in ribonucleocapsid formation and viral genome replication. In order to elucidate its functions, interaction partners of the SARS-CoV-2 N protein in human cells were identified via affinity purification and mass spectrometry. We identified 160 cellular proteins as interaction partners of the SARS-CoV-2 N protein in HEK293T and/or Calu-3 cells. Functional analysis revealed strong enrichment for ribosome biogenesis and RNA-associated processes, including ribonucleoprotein complex biogenesis, ribosomal large and small subunits biogenesis, RNA binding, catalysis, translation and transcription. Proteins related to virus defence responses, including MOV10, EIF2AK2, TRIM25, G3BP1, ZC3HAV1 and ZCCHC3 were also identified in the N protein interactome. This study comprehensively profiled the viral–host interactome of the SARS-CoV-2 N protein in human cells, and the findings provide the basis for further studies on the pathogenesis and antiviral strategies for this emerging infection.

## 1. Introduction

By July 2021, the global pandemic caused by COVID-19 had resulted in 190,860,860 confirmed cases and more than 4,101,400 deaths, according to WHO data [1]. The disease is caused by a novel strain of coronavirus named severe acute respiratory syndrome coronavirus-2 (SARS-CoV-2), which contains four structural proteins; spike (S) glycoprotein, envelope (E) protein, membrane (M) protein and nucleocapsid (N) protein. These proteins share high levels of sequence similarity with corresponding sequences in SARS-CoV and MERS-CoV, which may reflect a common mechanism of pathogenesis [2]. The N protein of this virus is the most abundant protein, and it performs multiple functions during viral infection. The primary function of the SARS-CoV-2 N proteins is to package the viral genome into ribonucleocapsid (RNP). It is also involved in viral replicase components to modulate viral RNA transcription and replication [3]. 

It is well known that virus replication relies heavily on the complex protein–protein interaction (PPI) network formed by specific viral–host interactions. In response, host cells employ antiviral defences through the PPI network. Several individual proteomic studies have mapped the PPI network between SARS-CoV-2 proteins and human proteins, extending our knowledge of viral pathogenesis [4,5,6,7,8]. Given the multiple functions of the N protein, and its high expression during SARS-CoV-2 infections, we applied a systematic affinity tag purification and mass spectrometry (AP-MS) approach to identify host proteins that interact with the nucleocapsid protein of SARS-CoV-2. A total of 160 high-confidence human proteins with various biological functions were identified in HEK293T and/or Calu-3 cells. Our results present a comprehensive interaction landscape for the N protein and human proteins, and provide valuable clues for understanding the pathogenic mechanisms of the N protein inside human cells.

## 2. Results

### 2.1. Affinity Purification of SARS-CoV-2 N Protein in HEK293T and Calu-3 Cells

**Figure 1:**
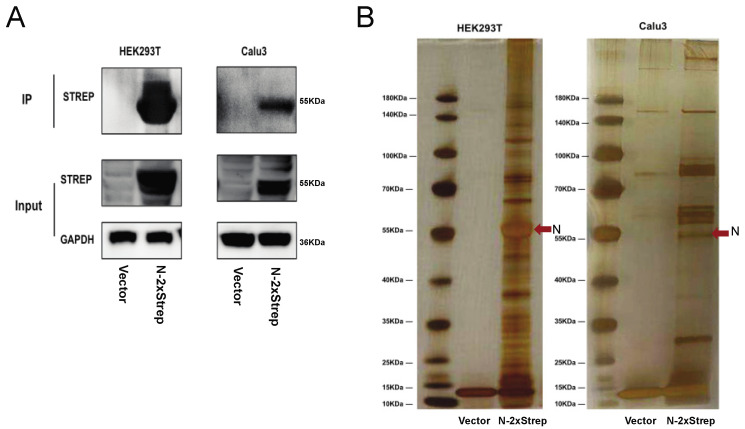
Identification of potential cellular proteins that interact with SARS-CoV-2 N protein by transient transfection and affinity purification from HEK293T and Calu-3 cells. (**A**) Detection of 2x Strep-tagged N protein in lysates and immunoprecipitates by western blotting with anti-Strep antibodies. (**B**) Protein complexes immunoprecipitated using Strep beads separated by SDS-PAGE and subjected to silver staining.

In order to explore the potential partners interacting with the N protein, a 2× Strep tag sequence was appended to the C-terminus of the SARS-CoV-2 N protein-coding sequence, and plasmids were transfected into HEK293T and Calu-3 cells. Tagged proteins were affinity purified via a coimmunoprecipitation approach. The SARS-CoV-2 N-Strep fusion protein was found to be highly expressed in both HEK 293T and Calu-3 cells (Figure 1A). Silver staining showed the specific enrichment of the SARS-CoV-2 N protein and its associated factors (Figure 1B).

### 2.2. Identification of Host Factors That Interact with SARS-CoV-2 N Protein in HEK293T and Calu-3 Cells

**Figure 2:**
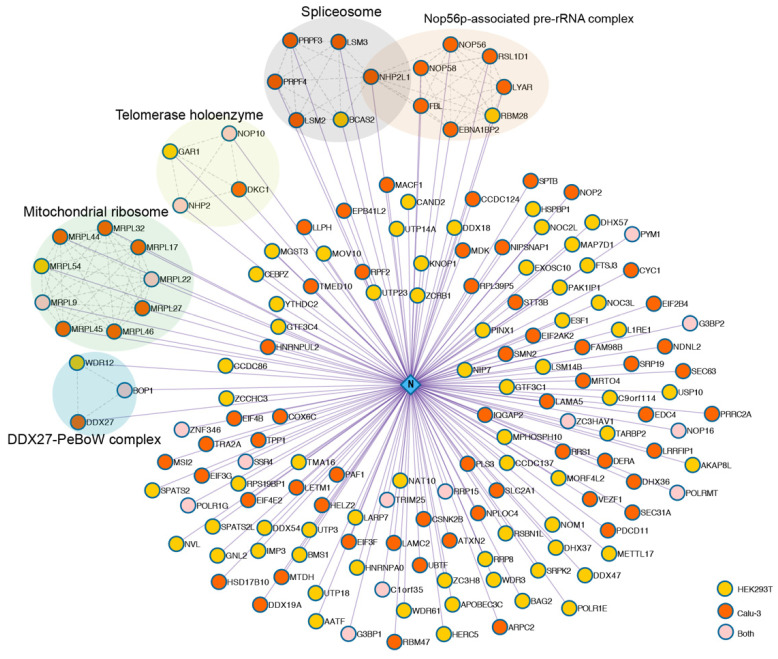
SARS-CoV-2 N-human protein–protein interaction network. An interaction network (Cytoscape) between host factors and N proteins was generated. Interactomes in two cell lines were integrated (yellow nodes = interactors in HEK293T cells, orange nodes = interactors in Calu-3 cells, pink nodes = common interactors in both cell types). Curated host–host protein interactions from the CORUM and STRING databases are displayed as coloured subnetworks. Select human protein–protein complexes that are represented by at least three nodes are labelled.

The N-interacting proteins were analysed by liquid chromatography-MS (LC-MS). We obtained 1347 and 2549 proteins in HEK293T and Calu-3 cells, respectively (Table S1). In order to eliminate false positive interactions, strict screening criteria were set, including the following: (1) fold change >8; (2) significance analysis of interactome (SAINT) [9] score >0.9; (3) mass spectrometry interaction statistics (MiST) [10] score >0.85; (4) protein detection frequency <30% in AP-MS Strep control datasets obtained from the contaminant repository for affinity purification (CRAPome) [11] database. Finally, 160 unique high-confidence protein interactions (85 in HEK293Tcells, 92 in Calu-3 cells) were identified and visualised as a protein–protein interaction (PPI) network (Figure 2, Table S1). Several protein components involved in specific protein families or pathways were identified and displayed as coloured subnetworks, including mitochondrial ribosome, spliceosome, nop56p-associated pre-rRNA complex, telomerase holoenzyme and DDX27-PeBow complex, all of which are closely associated with ribosome biogenesis and RNA processes in eukaryotes. Spliceosome components that bind directly to nucleocapsid proteins of other coronaviruses have been reported [12,13]. Notably, recent proteomics research revealed 25 spliceosome components upregulated following SARS-CoV-2 infection, and inhibition of splicing could prevent viral replication [14]. Thus, splicing is an essential pathway for SARS-CoV-2 replication.

We identified 17 shared human proteins in HEK293T and Calu-3 cells (Table 1), including ribosome biogenesis proteins BOP1, MRPL22, RRP15, NOP16, NOP10 and NHP2; RNA catalytic proteins POLRMT and POLR1G; virus defence proteins G3BP1, G3BP2, ZC3HAV1 and TRIM25, and various others. Five of the overlapping proteins, G3BP1, G3BP2, BOP1, ZNF346 and TRIM25 have been present in previous studies [4,5,6,7,8,15]. TRIM25 is an E3 ubiquitin ligase, which activates the type I interferon (IFN) pathway through the ubiquitination of RIG-I and ZC3HAV1 [16,17]. ZC3HAV1 can significantly restrict virus replication [18]. TRIM25 was also found to interact with the N protein of SARS-CoV and MERS-CoV. The N protein can impede RIG-I ubiquitination and activation to inhibit the production of IFN by interacting with TRIM25 [19,20]. Since SARS-CoV-2 is more than 82% identical at the genome level to SARS-CoV [21], it may evade the host’s innate immune response through a similar mechanism. 

### 2.3. Functional Annotation of Proteins in the SARS-CoV-2 N Protein Interactome

**Figure 3:**
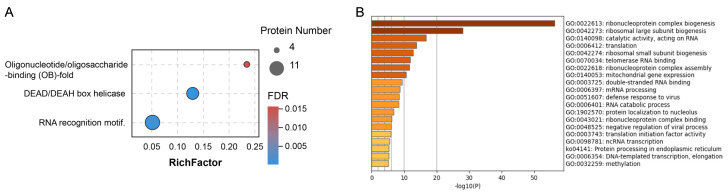
Functional analysis of the SARS-CoV-2 N interactome. (**A**) Bubble visualisation of enriched protein domains among SARS-CoV-2 N interactome members (FDR < 0.05). The colour of the dots represents the false discovery rate (FDR) value for each enriched domain term, and the size represents the percentage of proteins enriched. (**B**) Bar graph of the top 20 enriched functional (GO and KEGG) terms across the SARS-CoV-2 N interactome, coloured by −log10 (*p*-value).

To further explore the functions of these host cellular proteins, gene set enrichment analysis was performed using Pfam, Gene Ontology (GO) and Kyoto Encyclopedia of Genes and Genomes (KEGG). We observed three enriched protein domains including RNA recognition motif, DEAD/DEAH box (DDX) helicase and oligonucleotide/oligosaccharide-binding fold (Figure 3A, Table S2). A variety of DDX helicase family members (DDX54, DDX18, DDX27, DHX36, DHX37, DDX47, DDX19A, YTHDC2 and DHX57) were observed in our AP-MS interactome. DDXs have been described as key players in viral replication since they can positively or negatively modulate innate immunity and viral proliferation at different levels [22]. RNA recognition motif was the most highly-represented domain among host proteins, present in 11 host proteins including LARP7, RBM28, RBM47, EIF4B, EIF3G, MSI2, ZCRB1, G3BP1, G3BP2, HNRNPA0 and TRA2A (Table S2). This may imply that interactors of the N protein preferentially bind with RNA.

Functional analysis, also revealed strong enrichment for ribosome biogenesis and RNA-associated processes, including ribonucleoprotein complex biogenesis, ribosomal large and small subunits biogenesis, RNA binding, catalysis, translation and transcription (Figure 3B, Table S2). Consistent with these pathway terms, all domains of the SARS-CoV-2 N protein are predicted to bind RNA [23]. Notably, we also observed virus defence responses enriched in the N protein interactome. Several antiviral proteins such as MOV10, EIF2AK2, TRIM25, G3BP1, ZC3HAV1 and ZCCHC3 were included in this pathway (Table S2). Furthermore, N protein-associated host factors also participate in protein processing in the endoplasmic reticulum (ER). The transcription and replication of SARS-CoV-2 vRNA occurs in double membrane vesicles (DMVs) derived from ER [24]. Proteins involved in these biological processes may play a role in the N protein mediated regulation of viral genome replication and vRNP assembly.

### 2.4. Comparison of the N Protein Interactome with Other SARS-CoV-2-Induced Proteomes and Transcriptomes

**Figure 4:**
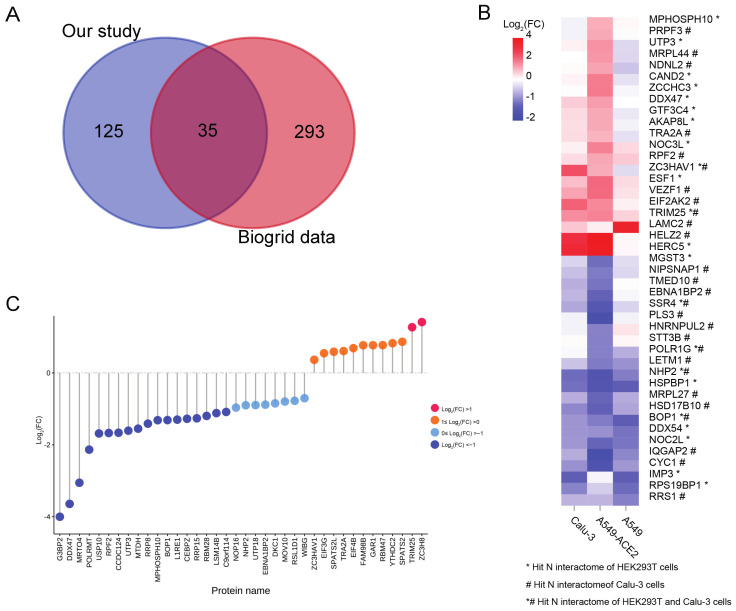
Overlapping information between the N protein interactome and other SARS-CoV-2-induced proteomes and transcriptomes. (**A**). Venn diagram showing the overlapping N protein interactions between our study and data obtained from the BioGRID [25] database. (**B**). Heatmap showing the variation in 43 genes overlapping between the N protein interactome of our study and previous SARS-CoV-2 induced transcriptome datasets [31]. (**C**). Point bar graph showing the expression of overlapped proteins between the N protein interactions and SARS-CoV-2 induced RNA-binding proteome [30]. FC, fold change.

To comprehensively survey the potential repertoire of host cellular factors that interact with the SARS-CoV-2 N protein, we obtained the currently known N protein interactions from the BioGRID database [25], and compared it with our data. Our AP-MS analysis shares ~22% overlap (35 hits) with previous studies (Figure 4A). Most of the overlapping hits were present in AP-MS studies and involved in ribosome biogenesis and RNA process (for example FBL, BMS1, NOP56, BOP1, WDR12, DHX37 and DDX54) and immune response (for example, MOV10, EIF2AK2, TARBP2, TRIM25, HERC5 and ZCCHC3) (Table S3). It is important to note that two stress granule proteins, G3BP1 and G3BP2, were most consistently identified in other studies [4,5,6,7,8,15,26,27,28,29]. G3BP1 and G3BP2 are downregulated during SARS-CoV-2 infection, and inhibition of stress granule formation by the N protein indicates that the N protein is involved in suppressing the host immune response to favour virus replication [5,30].

To further analyse the variation in proteins interacting with the N protein during SARS-CoV-2 infection, we compared our N protein interactome with host transcriptional response datasets obtained following SARS-CoV-2 infection [31]. Forty-three proteins in our dataset were significantly altered in the transcriptome dataset (fold change >2 and FDR <0.05 in at least one cell lines), while the variation in transcripts differed between cell lines (Figure 4B, Table S3). Nineteen genes were downregulated following SARS-CoV-2 infection in all three cell lines. Meanwhile, 10 genes, including three antiviral factors, TRIM25, HERC5 and EIF2AK2, were upregulated after SARS-CoV-2 infection. Since our N protein interactome was enriched in RNA-related processes, we next compared our dataset with recently reported SARS-CoV-2-induced RNA-binding proteome data [30]. Thirty-eight RNA-binding proteins were present in our N protein interactome dataset, 12 of which were upregulated after SARS-CoV-2 infection, including antiviral factors TRIM25 and ZC3HAV1, consistent with host transcriptional responses in Calu-3 cells and A549-ACE2 cells (Figure 4B,C, Table S3). 

## 3. Discussion

In the present study, we identified 160 high-confidence N-interacting host factors in HEK293T and/or Calu-3 cells via a proteomic approach. The PPIs of the N protein differed between the two cell lines, and only 17 shared human proteins were identified in both HEK293T and Calu-3 cells. Similarly, recent transcriptome studies revealed that host transcriptional responses to SARS-CoV-2 infection in different cell lines were markedly different [31,32]. Strong differences between the efficiency and productivity of SARS-CoV-2 infection across cell lines may explain the above phenomenon. For example, HEK293T cells were relatively non-permissive to SARS-CoV-2 replication due to the low expression of the viral receptor ACE2, compared with Calu-3 cells [33].

Interestingly, we also identified several mitochondrial proteins in the N protein interactome. SARS-CoV-2 could evade the mitochondrial production of interferons through its N protein [34]. The nucleocapsid protein induced apoptosis has been observed in transmissible gastroenteritis coronavirus (TGEV), porcine epidemic diarrhoea virus (PEDV) and SARS-CoV virus [35,36,37]. The cleavage of the N protein by effector caspases induces the intrinsic apoptotic pathway during SARS-CoV infection [37]. Additionally, the destruction of the mitochondrial structure, such as loss of the mitochondrial matrix and the ridges and rupture of the outer membrane, has been observed in the electron microscopy of COVID-19 patients’ tissue [38]; although it has been proved that SARS-CoV-2 viral RNAs, such as S, M, E, N and various others, were enriched in the host mitochondria [39]. There is still no evidence that N proteins affect the structure and function of mitochondria via directly interacting with mitochondrial proteins. Thus, the role of the interaction of N with these mitochondria proteins is either a false positive or could indicate a novel interaction with the mitochondria, and that more experiments are needed to validate this result.

Comparison of our data and previous interactomic studies showed 35 overlapping interactions. Several immune response proteins, such as MOV10, EIF2AK2, TARBP2, TRIM25, G3BP1, HERC5 and ZCCHC3 were included in the dataset. Ubiquitin-like protein ISG15 is an interferon-induced protein and is known to have a direct antiviral effect on a wide spectrum of virus families [40]. HERC5 and TRIM25 are major E3 ligases for ISG15 conjugation, and mediate ISGylates to activate the antiviral response [41,42]. EIF2AK2 has a broad antiviral spectrum and could upregulate type-1 interferon production via the integrated stress response [42,43,44]. It has been proved that the N protein can significantly repress the activation of IFN signalling [34].

Overall, the number of overlapping proteins between different datasets was limited. A couple of reasons may explain this. Firstly, the experimental methods, such as cell line selection, choice of affinity tag and screening criteria for high-confidence PPIs were different in each AP-MS experiment. Transcriptome studies have revealed different host transcriptional responses to SARS-CoV-2 infection in different cell lines [31,32]. Additionally, nonspecific interactions could be observed in AP-MS experiments, and each tag has its own specific background protein profile [45]. Secondly, AP-MS is not entirely suitable for the detection of protein complexes with weak affinity interactions or transient interactions, which might be lost during stringent rinsing procedures [46].

While differences were expected between the specific interactions of different studies, there is an overall consistency at the functional level. Functional analysis revealed strong enrichment for the RNA process, and the translation and transcription process. These data strongly suggest that the N protein plays an important role in viral transcription, translation and genome replication. Recent studies have pointed out that the SARS-CoV-2 N protein is capable of forming or regulating biomolecular condensates by interacting with RNA and key host cell proteins [47,48]. These structures are considered to play important roles in viral replication and assembly [49]. Thus, we compared our dataset with recently reported SARS-CoV-2-induced RNA-binding proteome and host transcriptional response datasets, and many proteins overlapped with these two datasets. These results indirectly confirmed that some of the proteins in our interactome are involved in biological processes during SARS-CoV-2 infection. 

SARS-CoV-2 N protein may be an important virulence factor since it not only plays critical roles in virus replication, transcription and translation but has also been proposed to perform roles in modulating the host cellular machinery [50]. Our current work provides an overview of potential host proteins that interact with the N protein. Although N protein interactomes differed between the two cell lines, the overlapping PPIs between different interactomes are more likely to represent physiologically relevant interactions. The identified PPIs provide valuable information at the molecular level for the virus replication cycle and pathogenesis. The findings also reveal potential druggable targets that may assist the development of new antiviral drugs, or the repurposing of existing drugs. While the identified proteins were not verified in this study, their specific roles during SARS-CoV-2 infection should be further explored in the future.

## 4. Materials and Methods

### 4.1. Cell Culture

Human embryonic kidney (HEK293T) cells (ATCC CRL-3216) and human lung adenocarcinoma (Calu-3) cells (ATCC HTB-55) were cultured in Dulbecco’s modified Eagle’s medium (DMEM; Gibco BRL, Grand Island, NY, USA) or minimum essential medium (MEM; Gibco BRL) supplemented with 10% foetal bovine serum (FBS; Gibco BRL), 1% penicillin–streptomycin (Gibco BRL), 1% sodium pyruvate (Gibco BRL) and 1% non-essential amino acids (Gibco BRL) at 37 °C in a humidified 5% CO_2_ incubator. 

### 4.2. Plasmids and Transfection

The coding sequence of SARS-CoV-2 N (GeneID: 43740575) was cloned into the mammalian expression vector pcDNA3.1 (+) harbouring a C-terminal 2xStrep II affinity tag. The pcDNA3.1 (+)-2xStrep vector alone served as a control. 1–1.2 × 10^7^ HEK293T and Calu-3 cells were plated in 15 cm dishes and allowed to adhere overnight prior to transfection with 15 μg plasmids using FuGENE HD transfection reagent (Promega Corporation, Madison, WI, USA). At least three independent biological replicates were performed in each cell line. 

### 4.3. Anti-Strep Tag Affinity Purification

At 48 h post-transfection, cells were dissociated from the plate surface with 1× phosphate-buffered saline (PBS) containing 10 mM EDTA, subsequently washed with cold 1× PBS, and lysed in IP buffer (50 mM Tris-HCl pH 7.4, 150 mM NaCl, 1 mM EDTA) supplemented with 0.5% Nonidet P40 substitute (NP-40; Solarbio, Beijing, China) and cOmplete mini EDTA-free protease and PhosSTOP phosphatase inhibitor cocktails (Roche, Bransburg, NJ, USA). Cells were lysed on ice for 30 min then cleared by centrifugation at 17,000× *g* for 10 min at 4 °C. After centrifugation, the supernatant was incubated with 30 μL Strep-Tactin Sepharose beads (IBA Lifesciences, Göttingen, Germany) diluted in IP Buffer for 2 h. Beads were then washed three times with 1 mL IP buffer supplemented with 0.05% NP-40 and transferred to a new tube with a final wash in 1 mL IP buffer. Proteins were eluted by agitating beads in 40 μL IP buffer supplemented with 2.5 mM D-desthiobiotin (IBA Lifesciences) on a vortex mixer at room temperature for 30 min. We reserved 10% of each eluate for western blotting and silver staining. The remaining eluate was removed for mass spectrometry (MS). 

### 4.4. Peptide Preparation 

Eluates were incubated in 10 mM dithiothreitol (DTT) for 45 min and subsequently alkylated with 30 mM iodoacetamide (IAA) for 30 min at room temperature in the dark. IAA was quenched by DTT (20 mM final concentration). Samples were then cleaned up using four volumes of acetone for precipitation, and pellets were resuspended in 50 mM ammonium bicarbonate. Trypsin (Promega) was added to samples at a ratio of 1:50 (trypsin–protein) and incubated overnight at 37 °C to digest protein. Peptides were acidified with fluoroacetic acid (FA) and desalted using a C18 desalting column equilibrated in 200 mL acetonitrile (ACN), then twice with 200 mL 60% ACN followed by 200 mL 0.1% FA. Samples were loaded onto the C18 column, washed three times with washing buffer (0.1% FA, 2% ACN), then eluted with 60% ACN. Eluents were collected and lyophilised in a vacuum lyophilizer (Labconco, Kansas City, MO, USA) before LC-MS/MS analysis.

### 4.5. Protein Identification by LC-MS/MS

Samples were resuspended in 2% ACN and 0.1% FA and separated by nano liquid chromatography (LC)-MS/MS using an UltiMate 3000 RSLCnano system (Thermo Scientific, Grand Island, NY, USA)) at a flow rate of 400 nL/min. Solvent A was 2% ACN and 0.1% FA, and solvent B was 98% ACN and 0.1% FA. Gradient elution was performed at 50 °C using linear gradients of 120 min as follows: 1–4 min 3% (*v*/*v*) B, 4–6 min 3% to 5% (*v*/*v*) B, 6–70 min 5% to 15% (*v*/*v*) B, 70–90 min 15% to 30% (*v*/*v*) B, 90–100 min 30% to 80% (*v*/*v*) B, 100–110 min 80% (*v*/*v*) B, 110–120 min 3% (*v*/*v*) B. Eluted peptides were analysed using a Q Exactive HF-X instrument (Thermo Scientific) to acquire MS spectra at a resolution of 120,000 FWHM with a mass range of 300–1500 *m/z* and an AGC target of 3E6. The top 20 precursors were then fragmented by HCD with a collision energy of ~32% NCE and MS2 spectra were acquired at a resolution of 45,000 FWHM.

Raw LC-MS/MS data were analysed by MaxQuant (version 1.6.2.10) against a database containing the UniProt *Homo sapiens* protein sequences (192,321 sequences, updated on 2 July 2020) and the SARS-CoV-2 N protein sequences. All peptide and protein identifications were filtered by false discovery rate (FDR) <1%. 

### 4.6. Protein-Protein Interaction (PPI) and Enrichment Analyses

Proteomic data were scored with the MiST [10] and SAINT [9] scoring algorithms using spectral counts as the quantifying feature. The selected high-confidence PPIs were visualised by Cytoscape (version 3.8.0). Metascape [51] and DAVID [52] databases were used for gene annotation, visualisation and enrichment analysis. Terms from GO, specifically molecular function (MF) and biological process (BP) categories, as well as Pfam, KEGG and the comprehensive resource of mammalian protein complexes (CORUM) were considered. CORUM complexes were analysed with STRING [53] (version 111.0, https://string-db.org/cgi/input.pl, accessed on 20 July 2021) and visualised in the PPI network. Statistical significance of each gene function category was scored using the standard accumulative hypergeometric probability function. Enrichment factors were calculated and used for filtering. Remaining significant terms were further clustered into groups based on similarities measured by Kappa statistics, similar to the method used in DAVID. Terms with *p*-value < 0.05 or FDR < 0.05 were considered significantly enriched.

## Figures and Tables

**Table 1:** Overlapping SARS-CoV-2 N protein interactors in HEK293T and Calu-3 cell lines.

Gene ID	Gene Symbol	Protein Name	Biological Process (GO)
23246	BOP1	Block of proliferation 1 protein	Endonucleolytic cleavage involved in rRNA processing
79169	C1orf35	Multiple myeloma tumour-associated protein 2	Neutrophil-mediated immunity
5442	POLR1G	DNA-directed RNA polymerase I subunit G	Positive regulation of gene expression
10146	G3BP1	Ras GTPase-activating protein-binding protein 1	Stress granule assembly
9908	G3BP2	Ras GTPase-activating protein-binding protein 2	Stress granule assembly
29093	MRPL22	Mitochondrial large ribosomal subunit protein uL22m	Ribosome assembly
65005	MRPL9	Mitochondrial ribosomal protein L9	Mitochondrial translational elongation
55651	NHP2	H/ACA ribonucleoprotein complex subunit 2	snRNA/rRNA pseudouridine synthesis
55505	NOP10	Nucleolar protein 10	snRNA pseudouridine synthesis
51491	NOP16	Nucleolar protein 16	Ribosome biogenesis
5442	POLRMT	DNA-directed RNA polymerase, mitochondria	Mitochondrial transcription
51018	RRP15	Ribosomal RNA-processing protein 15	Ribosomal large subunit biogenesis
6748	SSR4	Translocon-associated protein subunit delta	Protein processing in endoplasmic reticulum
7706	TRIM25	Tripartite motif containing 25	Ubiquitin E3 ligase and ISG15 E3 ligase
84305	PYM1	Partner of Y14 and mago	Positive regulation of translation
56829	ZC3HAV1	Zinc finger CCCH-type antiviral protein 1	Antiviral defence, Immunity, Innate immunity
23567	ZNF346	Zinc finger protein 346	Positive regulation of cell death

## Data Availability

All data generated or analyzed during this study are included in the Supplementary Materials.

## Supplementary Materials

The following are available online at https://www.mdpi.com/article/10.3390/pathogens10091155/s1, Table S1: Proteins identified by LC-MS/MS analysis and high-confidence PPIs of N protein interactome. Table S2: Functional enrichment of host factors. Table S3: Overlapping data between our N protein interactome and other SARS-CoV-2-induced proteomes and transcriptomes.
